# Supplementary figures and images for: Statistical methodologies for evaluation of the rate of persistence of Ebola virus in semen of male survivors in Sierra Leone
Source: PLoS One. 2022 Oct 5;17(10):e0274755. doi: 10.1371/journal.pone.0274755 (PMC9534448; doi:10.1371/journal.pone.0274755)

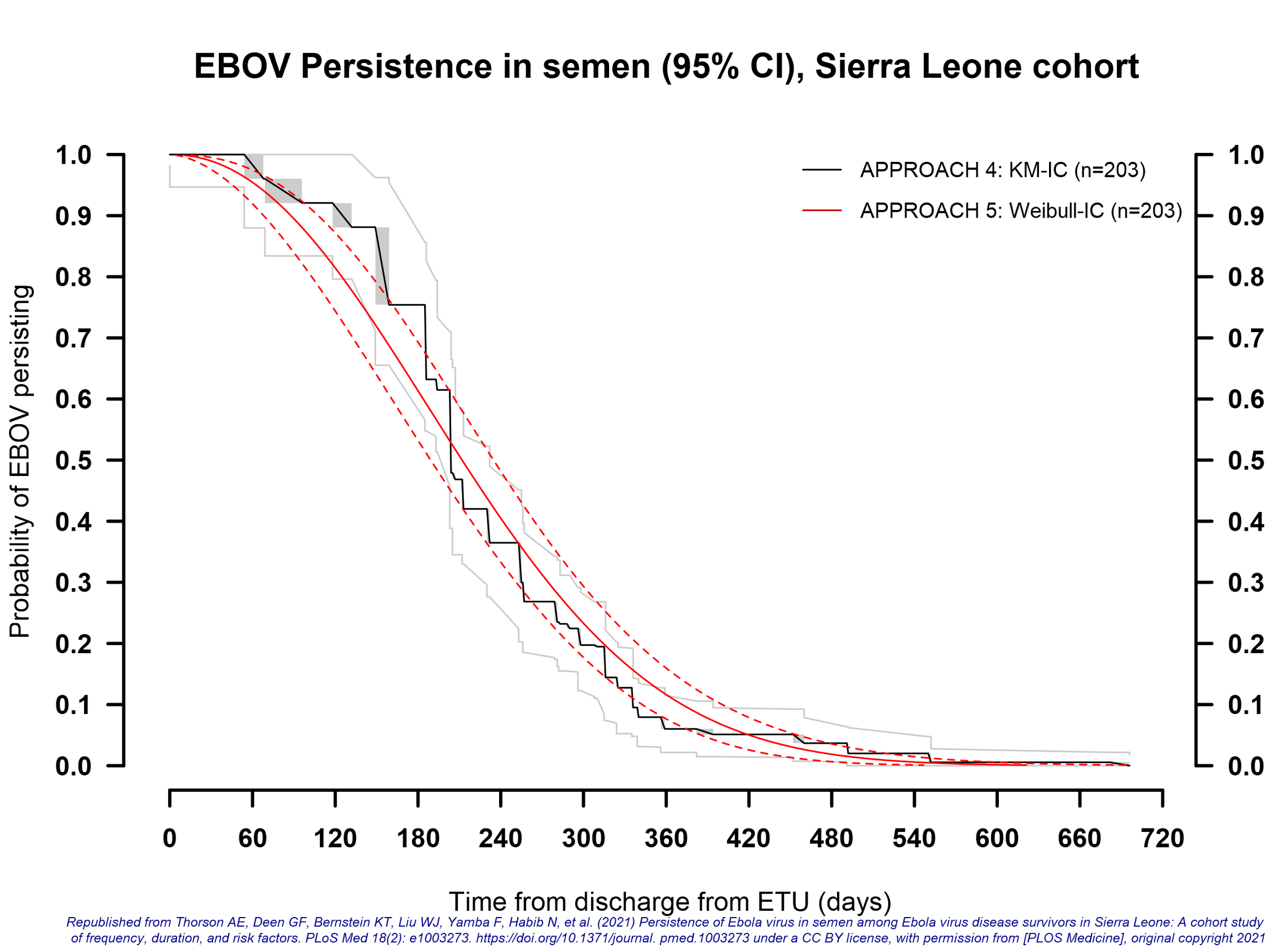

Supplement: S1 Fig — “Republished from [Thorson AE, Deen GF, Bernstein KT, Liu WJ, Yamba F, Habib N, et al. (2021) Persistence of Ebola virus in semen among Ebola virus disease survivors in Sierra Leone: A cohort study of frequency, duration, and risk factors. PLoS Med 18(2): e1003273. https://doi.org/10.1371/journal. pmed.1003273] under a CC BY license, with permission from [PLOS Medicine], original copyright [2021]”. (TIF) [file pone.0274755.s001.tif]
