# Supplementary material for: Statistical methodologies for evaluation of the rate of persistence of Ebola virus in semen of male survivors in Sierra Leone
Source: PLoS One. 2022 Oct 5;17(10):e0274755. doi: 10.1371/journal.pone.0274755 (PMC9534448; doi:10.1371/journal.pone.0274755)
Supplement: Attachment — Submitted filename: Habib et al notes.docx [file pone.0274755.s002.docx]

The authors of this manuscript present convincing evidence for use of statistical methods that consider interval censoring when examining persistence of EBOV in semen among SLEVPS participants. Their methods section does a good job introducing different statistical options along with their weaknesses. However, the writing in other sections of this paper, while grammatically correct, is often not written with clear arguments and supporting evidence. The introduction and discussion sections suffer in particular. It is unclear whether the authors are writing a methods tutorial paper with SLEVPS as a convenient example, if they are writing specifically to researchers who work with data like SLEVPS, or if they are trying to improve estimates of EBOV persistence in semen. I recommend that the authors think about what they are trying to communicate specifically with this manuscript and to whom. Once that is identified, I think it will be easier to resolve many of the issues.

- General notes:
  - Writing style often has a lot of fluff. Could generally be more to the point.
  - Authors have a tendency to make vague claims with little support.
  - I expected some examination of existing literature (or of mention of lack thereof) that has either used or failed to use appropriate statistical approaches to evaluating time to event data in this context. However, that context is not present in this manuscript. What are the mistakes people are making? How might this have affected previous estimation of EBOV persistence in semen? What do the authors findings mean in the broader context of EBOV persistence research?
- Abstract
  - Imprecise language in 31-32. While I think the intent is to compare the 2013-2016 EBOV outbreak to other EBOV outbreaks, it reads as a comparison between that outbreak and other outbreaks of any kind. This sentence as written at the beginning of the introduction (55-56) is slightly better but could still use some work.
  - Imprecise language in 31-32 (cont.). The authors state that the 2013-2016 outbreak was the "largest and most complex outbreak ever." While they clarify that "largest" means in terms of relative case numbers, there is no explanation of "most complex." This same issue appears in 55-56.
  - In general abstract could do a better job of succinctly and precisely presenting the rationale for the study/motivating problem, the methods considered for addressing that problem, the data used to examine those methods (population, sampling approach, sample size, etc), results, and conclusions.
- Introduction
  - [61-62] Vague descriptions. In which body fluids do we know virus persists, how "rapidly" does it disappear, how does this compare to the 3-month statistic cited in the next sentence?
  - [62-64] In what way were these data limited?
  - [65-66] This is an awkward way to introduce the problem/statistic under examination. Better to first present what you are trying to describe (time to undetectable EBOV in semen) and then introduce the approach that best fits that problem in an ideal scenario.
  - [108-110] It's not a bold claim to make that right censoring methods are more widely applied than interval or left-censoring methods, but I don't think that one can claim those methods are less widely known based off of the author's selection of literature. I would be surprised if any of my peers was completely unaware of these methods, but many researchers don't encounter situations where they should use them.
  - [113-114] Why not just list the software? The article cited here is 8 years old and I suspect that most of the software most commonly used (SAS, STATA, R, etc) support analysis with interval censored data.
- Methods
  - The methods themselves are well-described, but insight into the rationale for choosing this particular set of methods may be lacking.
  - [256-261] This explanation is correct but the equation is incorrect. The midpoint between l_2_ (time of last + visit) and t_2_ (time of first - visit) would be $l_{2}+\frac{\left( t_{2}-l_{2} \right)}{2}$. This error also occurs in Table 1.
  - [265-267] There is a solid statistical rationale for why midpoint imputation can underestimate the standard error. Bias when using midpoint imputation also has a solid foundation, but the consequences are more conceptually complex. I think the way this is reported is fine but would prefer to see these two biases discussed as separate ideas rather than in one long sentence.
  - [307] Would prefer to see NPLME KM written out. Appears in a heading and what it means is not written out before this point.
  - [308-309] Are these actually recent developments? The original Turnbull paper was published in the 70s and a quick google search shows that an R package accommodating these methods was published around 2010. Avoid making unsubstantiated or unclear claims.
  - [307-341, 368-386] While I am not opposed to more equation-based explanations in papers, the choice to provide this level of detail in these sections is somewhat puzzling. These seem like opportunities to explain the why these approaches may be preferred and conceptually how they work. Perhaps the approach used in these sections confuses me because it is unclear what audience the authors are intending to reach.
- Discussion
  - [450-451] One cannot say that Approach 4 is preferred simply because its estimates are "more precise and statistically unbiased." Inference is limited to the data at hand in this project. Thus, one cannot know the true unbiased estimate, and increased precision doesn't necessarily indicate an improvement. An example of this issue – midpoint imputation has very precise estimates at 50% and 75% -- is already baked into this manuscript. The authors need to consider and articulate their arguments clearly. They have outlined reasons why we would expect approaches 1-3 to generate estimates that poorly resemble the truth, and how approaches 4-5 account for the limitations of 1-3. Lean on that argument here.
  - [454-458] Underestimated relative to what?
- Conclusions
  - [518-519] First sentence of conclusions could be deleted.
  - Both in the introduction and discussion, I expect some examination of existing literature (or of mention of lack thereof) that has either used or failed to use a strong statistical approach to evaluating time to event data in this context. However, that context is not present in this manuscript. What are the mistakes people are making? How might this have affected previous estimation of EBOV persistence in semen? Etc.
