# Supplementary material for: Statistical methodologies for evaluation of the rate of persistence of Ebola virus in semen of male survivors in Sierra Leone
Source: PLoS One. 2022 Oct 5;17(10):e0274755. doi: 10.1371/journal.pone.0274755 (PMC9534448; doi:10.1371/journal.pone.0274755)
Supplement: Attachment — Submitted filename: Response to Reviewres_PLOS One_Review_24Aug2021.docx [file pone.0274755.s003.docx]

**Dr. Ndema Habib**

**The Department of Sexual and Reproductive Health Research (SRH),**

**The World Health Organization (WHO), Geneva, Switzerland**

**Telephone (office): +41227914516; e-mail: habibn@who.int**

**Dr. Joerg Heber**

**Editor-in-Chief, *PLOS ONE***

**04-July-2021**

We are also pleased to respond to the very important reviewers’ comments as follows:

**Reviewer #1:** The authors of this manuscript present convincing evidence for use of statistical methods that consider interval censoring when examining persistence of EBOV in semen among SLEVPS participants. Their methods section does a good job introducing different statistical options along with their weaknesses. However, the writing in other sections of this paper, while grammatically correct, is often not written with clear arguments and supporting evidence. The introduction and discussion sections suffer in particular. It is unclear whether the authors are writing a methods tutorial paper with SLEVPS as a convenient example, if they are writing specifically to researchers who work with data like SLEVPS, or if they are trying to improve estimates of EBOV persistence in semen. I recommend that the authors think about what they are trying to communicate specifically with this manuscript and to whom. Once that is identified, I think it will be easier to resolve many of the issues. Full comments in attached file.

**AUTHORS RESPONSE:** we are writing specifically to researchers who work on persistence estimation of Ebola in body fluids, like is the case for the SLEVPS, however these methodologies may be applicable to other areas facing similar study design challenges.

**GENERAL NOTES**

**COMMENT 1:** Writing style often has a lot of fluff. Could generally be more to the point.

**AUTHORS RESPONSE:** Manuscript has been revised to maximize clarity.

**COMMENT 2:** Authors have a tendency to make vague claims with little support.

**AUTHORS RESPONSE:** We have revised to ensure that claims are supported by evidence.

**COMMENT 3:** I expected some examination of existing literature (or of mention of lack thereof) that has either used or failed to use appropriate statistical approaches to evaluating time to event data in this context. However, that context is not present in this manuscript. What are the mistakes people are making? How might this have affected previous estimation of EBOV persistence in semen? What do the authors findings mean in the broader context of EBOV persistence research?

**AUTHORS RESPONSE:** We have added examination of literature in the introduction section lines 142-157. Many of the Ebola persistence studies are small in size and a good number involve case studies that are mostly descriptive. reporting the maximum duration of persistence. We found only one study (Sissoko et al 2016) that used the time series approach in the determination of persistence, and another (Subtil et al, 2017) that acknowledged the interval censored nature of the semen EBOV persistence data and used the appropriate methods. However no indepth description on how they determined the lower and upper bounds for the event of interest in the context of left- and interval censored events. Our paper seems to be among the first to describe thoroughly all aspects of the study design of EBOV persistence, as well as on how parameters can be estimated both in the context of non-parametric and parametric survival modelling. In the broader context our paper seeks to demonstrate the issues to consider designwise and analysis-wise while estimating persistence and proposes survival analyses techniques that take into account the interval nature of the data to accomodate the three types of censoring. In the presence of censoring, the survival techniques are still the most suited for assessment of persistence of EBOV in semen of survivors.

**ABSTRACT**

**COMMENT 4:** The methods themselves are well-described, but insight into the rationale for choosing this particular set of methods may be lacking.

**AUTHORS RESPONSE:** We have included rationale on the RC and IC methods on lines 237-242, and for the choice behind the Weibull parametric distribution on 388-394.

**COMMENT 5:** [256-261] This explanation is correct but the equation is incorrect. The midpoint between l2 (time of last + visit) and t2 (time of first - visit) would be . This error also occurs in Table 1.

$$l_{2}+\left( \frac{{t_{2}-l}_{2}}{2} \right)=\frac{\boldsymbol{l}_{\boldsymbol{2}}\boldsymbol{+}\boldsymbol{t}_{\boldsymbol{2}}}{\boldsymbol{2}}$$

**AUTHORS RESPONSE:** " Counting from time 0, the time in its most simplified form.

To conserve space, we have decided to keep the simplified original form of the equation. "

**COMMENT 6:** [265-267] There is a solid statistical rationale for why midpoint imputation can underestimate the standard error. Bias when using midpoint imputation also has a solid foundation, but the consequences are more conceptually complex. I think the way this is reported is fine but would prefer to see these two biases discussed as separate ideas rather than in one long sentence.

**AUTHORS RESPONSE:** We have separated these into two statements (lines 308-311)

**COMMENT 7:** [ [307] Would prefer to see NPLME KM written out. Appears in a heading and what it means is not written out before this point.

**AUTHORS RESPONSE:** the NPMLE has been written out in full on line 278. We have also written in out in full in the heading (line 349-351)

**COMMENT 8:** [  [308-309] Are these actually recent developments? The original Turnbull paper was published in the 70s and a quick google search shows that an R package accommodating these methods was published around 2010. Avoid making unsubstantiated or unclear claims.

**AUTHORS RESPONSE:** We have omitted the word "recent" from the statement.

**COMMENT 9:** [  [307-341, 368-386] While I am not opposed to more equation-based explanations in papers, the choice to provide this level of detail in these sections is somewhat puzzling. These seem like opportunities to explain the why these approaches may be preferred and conceptually how they work. Perhaps the approach used in these sections confuses me because it is unclear what audience the authors are intending to reach.

**AUTHORS RESPONSE:** We still think it is important to convey this information to readers so as to also enable those with interest in understanding the theoretical background as well as, in other cases to get an idea as to how the estimates were derived from the formulae.

**DISCUSSION**

**COMMENT 10:**  [450-451] One cannot say that Approach 4 is preferred simply because its estimates are "more precise and statistically unbiased." Inference is limited to the data at hand in this project. Thus, one cannot know the true unbiased estimate, and increased precision doesn't necessarily indicate an improvement. An example of this issue – midpoint imputation has very precise estimates at 50% and 75% -- is already baked into this manuscript. The authors need to consider and articulate their arguments clearly. They have outlined reasons why we would expect approaches 1-3 to generate estimates that poorly resemble the truth, and how approaches 4-5 account for the limitations of 1-3. Lean on that argument here.

**AUTHORS RESPONSE:** We have revised text accordingly.

**COMMENT 11:** [454-458] Underestimated relative to what?

**AUTHORS RESPONSE:** We have revised text. Relative to KM-IC curve (lines 519-520)

**Reviewer #2:** The authors present an interesting paper that describes different approaches to analysis of time to event data subject to different kinds of censoring. This endeavour deserves merit, and they explain very nicely the different approaches and show their results using a data set with information about traces of the Ebola virus in semen.

**AUTHORS RESPONSE:** We thank the reviewer for this motivating comment

**MAJOR ISSUES**

**COMMENT 1:** It is unclear what the main aim of using these models is. Should individual out-of-sample predictions be possible in order to advise future Ebola survivors? Or does the main interest lie in observing the mean or median behaviour (or the percentiles) of the population of Ebola survivors?

**AUTHORS RESPONSE:** The main interest is in describing the performance of these models, and commenting on the differences in the estimation of the cumulaive rate of persistence distribution as well as and percentile estimates using SLEVPS sample data.

**COMMENT 2:** This point is closely related to the former point and actually the most important part of my review: The authors can only describe the different estimated survival curves/percentiles/standard errors. But it is impossible to say which of the models is best, because the authors fail to define criteria which can be used to measure the model quality. At several places the authors claim that one or the other model is unbiased, but they cannot say this without a criterion for model quality. They sometimes describe that a model leads to more precise estimates in the sense of having smaller confidence intervals, but this does not necessarily mean that the respective model is correct.

**AUTHORS RESPONSE:** We have revised manuscript to compare the strength and the limitation of each of the approaches to estimating persistence.

**COMMENT 3:** Depending on the main goal of fitting these models (see point 1), I suggest the following changes: If the main goal is prediction, the authors should predefine criteria for predictive model choice such as proper scoring rules (e.g. the Brier score) that can take into account both sharpness (precision) and calibration of a predictive model or judge the calibration alone. In addition, they should separate the data set in two parts, the training set and the test set, because otherwise the model fit will always be too good. Alternatively, they could use some kind of cross-validation.

If, however, the main aim of the models is parameter estimation/a description of the population, they should show some criteria for model fit/model choice such as some version of AIC/BIC/DIC or the likes that are appropriate for the models they use and allow for a somewhat independent comparison of the quality of the models. In addition, they could add a plot that shows the observed data, so that it is easier to judge.

If the authors don't want or cannot do this, they could alternatively give a restructured description of the different approaches and their advantages and disadvantages (e.g. distributional assumptions, number of parameters,...) and only show the results of the models as an addition at the and, but they should remove all judgement from the results part that is not justified (all about bias or the notion that a more precise confidence interval is always better).

**AUTHORS RESPONSE:** We have followed this latter suggestion to discuss more on how the five survival models selected function, the results of the fit and more on advantages and disadvantages. We have only discussed on the perfomance of the models based on the estimates they give.

**COMMENT 4:** It becomes already clear from the description of the approaches that approach 1 and 2 that take only right-censoring into account are not appropriate for the data set. Therefore, it is not at all surprising that the estimates are quite far away from the ones from the models for interval-censored data, this statement is a bit trivial. It is still nice to see, but it does not add much to the paper, while using relatively much space in the methods part. I recommend to shorten the explanation a bit and focus more on the other three methods.

**AUTHORS RESPONSE:** We have revised the reduced the description of the right-censored data and add more on the interval censored methods (lines 388-410)

**COMMENT 5:**The authors write in the introduction that there are only few possibilities regarding statistical software with ready-to-use functions for interval-censored data. This gave me the impression that the five selected approaches were among those few, but this is only partly true. Whereas the models could be fitted using existing SAS routines, the standard errors for percentiles obviously had to be computed/programmed by hand, and R plotting functions were used (do such possibilities not exist in SAS?) which makes the recommendations by far more difficult to use for non-statistician readers. The authors should maybe make it clearer in the introduction that their paper also does not provide an easy solution to the software problem.

**AUTHORS RESPONSE:** Thanks for highlighting this. We have included this in the introduction (lines 137-141)

**COMMENT 6:** Again, closely related to the former point: The authors only use SAS. Can they please provide more information about functions in other software packages such as R and STATA, so that readers who don't have access to SAS can still use their recommendations?

**AUTHORS RESPONSE:** Yes. We have included R and STATA as among the statistical software programs that can also estimate IC survival data both in non-parametric and parametric way. (lines 408-410; 566-570) and integration of covariates in the IC regression models lines (561-571).

**COMMENT 7:** On page 15 the authors explain the approach for interval censoring and how the values for L and R are chosen in the different groups. For group P3 they say that R is chosen to be infinity. I wonder if that is equivalent to treating those values as right-censored or, respectively, how this works technically/computationally or what would change if some real, albeit very large, value would be used instead. Can the authors elaborate a bit more?

**AUTHORS RESPONSE:** We have added a sentence clarifying that for the right-censored individual the upper limit of 'infinity' is usually replaced by a missing value, which the program interprets it as right-censored value. (lines 328-330)

**COMMENT 8:** Would it be possible to integrate covariates in the models? And as the authors seem to think that the percentiles are of particular importance: Did they think about using some quantile regression model? I don't want the authors to actually use these models, but a short discussion would be informative.

**AUTHORS RESPONSE:** Yes, it is possible to integrate covariates in the semi-parametric PH IC models. We have brief paragraph that explains this and different statistical packages that can analyze this, also in the context of parametric modelling (lines 561-570)

**MINOR POINTS**

**COMMENT 9:** It would be helpful to have an explanatory graph at the beginning that shows the important time points (such as hospital discharge and the main timepoints used in the study), maybe along with the different types of censoring and trunkation. It would help to understand the situation and in consequence, parts of the explanations later on could be shortened a bit.

**AUTHORS RESPONSE:** Ythe important timepoints have been described in Figure 1 that was saved separately as S1 Fig.tiff.

**COMMENT 10:** On page 15 (top) the authors write that there is both left- and right-censoring present and then write "Therefore, the EBOV persistence data can be rightly be considered as interval-censored." This conclusion is wrong. Interval-censoring is not defined this way, but rather that the event of interest happens between two fixed time points. Actually, the authors continue with the correct definition, so I recommend to either delete or rewrite this sentence.

**AUTHORS RESPONSE:** We have revised lines 323-331 adequately describes the interval censoring.

**COMMENT 11:** The authors could consider moving Figure 3 to the appendix. It is already pretty clear from looking at Figure 2 that the three interval-censoring approaches yield comparable results and I therefore expected the confidence intervals to overlap. So, there is not much additional information.

**AUTHORS RESPONSE:** We have moved Figure 3 to the appendix

**COMMENT 12:** Is there a particular reason for choosing the 50th, 75th and 90th percentiles? Any clinical interpretation? If so, a short explanation would be nice.

**AUTHORS RESPONSE:** Percentiles of viral persistence in semen, provides the probability of the virus EBOV persisting beyond a certain time period, this being of clinical and public health importance as it helps in informing semen testing survivor programmes; as well as in formulating policies surrounding duration of use of certain preventive measures including sexual abstinence and condom use aimed at minimizing sexual transmission of the virus and therefore preventing possibility future outbreaks. Extreme upper tail viral persistence percentiles furthermore are important in understanding how long it takes following ETU discharge that a group of survivors slowest to clear EBOV become EBOV-free. We have added this explanation on lines 571-578.

We thank both reviewers for their time and for the very useful comments they provided.

Sincerely,

Ndema Habib, PhD

Statistician, The Department of Sexual and Reproductive Health Research (SRH), WHO, Geneva.
