# Supplementary material for: Statistical methodologies for evaluation of the rate of persistence of Ebola virus in semen of male survivors in Sierra Leone
Source: PLoS One. 2022 Oct 5;17(10):e0274755. doi: 10.1371/journal.pone.0274755 (PMC9534448; doi:10.1371/journal.pone.0274755)
Supplement: Attachment — Submitted filename: Response to Reviewers.docx [file pone.0274755.s004.docx]

**Dr. Ndema Habib**

**The Department of Sexual and Reproductive Health Research (SRH),**

**The World Health Organization (WHO), Geneva, Switzerland**

**Telephone (office): +41227914516; e-mail: habibn@who.int**

**Dr. Joerg Heber**

**Editor-in-Chief, *PLOS ONE***

**03-November-2021**

We are also pleased to respond to the very important reviewers’ comments as follows:

**Reviewer #1:** Review Comments to the Author

Thank you for so graciously considering and responding to our many comments. The revised manuscript is much improved and does a good job presenting the already solid research done by the authors.

**AUTHORS RESPONSE:** We appreciate your comment.

There are a few awkward sentences and grammatical errors (comma splices and run-on sentences are a common problem) at: 70-73, 76-79, 79-81, 82-85, 88-91, 92-93 & potentially other locations.

**AUTHORS RESPONSE:** Thank you. We have revised and corrected as per lines 71-74, 77-82, 83-86, 89-91, 92-93, as well as in other places.

[126-132] Side note (no changes/response requested) – left censoring is actually a big problem in many public health studies because of immortal time bias.

**AUTHORS RESPONSE:** This is well noted.

[126-141] Good paragraph

**AUTHORS RESPONSE:** Thank you for encouraging remark.

[142-157] Thank you for adding, this is helpful context.

**AUTHORS RESPONSE:** Noted. Thank you,

[Table 1] Unfortunately, equations in author response (and from my initial comment) were turned into an asterisk by (I suspect) the program that created my pdf, so I cannot fully evaluate author's response.

**AUTHORS RESPONSE:** In our last Response to reviewers’ letter, we prepared and attached separately authors’ responses in Word format as well. There, all the equations appear as they should have.

[530-534] It's interesting thinking about this problem with such a high proportion of left-censored participants.

**AUTHORS RESPONSE:** We thank the reviewer for this motivating comment

**Reviewer #2:**  Review Comments to the Author

Thank you for addressing my comments. I especially like the software information that is now given at several places.

My only recommendation is to go again through the manuscript with particular attention to grammar mistakes/funny expressions. Especially in the new or changed parts of the manuscript, there are several small mistakes, probably due to some haste in which the manuscript was rewritten.

**AUTHORS RESPONSE:** Thank you. We have now revised and corrected the grammatical errors.

We thank both reviewers for their time and for the very useful comments they provided.

Sincerely,

Ndema Habib, PhD

Statistician, The Department of Sexual and Reproductive Health Research (SRH), WHO, Geneva.
