# Supplementary material for: Statistical methodologies for evaluation of the rate of persistence of Ebola virus in semen of male survivors in Sierra Leone
Source: PLoS One. 2022 Oct 5;17(10):e0274755. doi: 10.1371/journal.pone.0274755 (PMC9534448; doi:10.1371/journal.pone.0274755)
Supplement: Attachment — Submitted filename: Comments -EBOV paper.docx [file pone.0274755.s005.docx]

**Title: Statistical methodologies for evaluation of the rate of**  **persistence of Ebola virus in semen of male survivors in**  **Sierra Leone**

**Comments:**

As I understand the authors want to study the survival period of EBOV (Ebola virus) in the semen of patients discharged from the hospital. In other words, it can be stated that the probability of occurrence of event X (X is negative EBOV) with the available censored data.

1. The total sample size of the study is only 203 cases and out of 203, 88 cases experienced the event (negative test of EBOV) before the day of recruitment. Only 115 cases were included for follow up study. At the same time follow up period was comparatively long i.e. till the last case was tested negative or withdrawn from the study; about 451 - 540 days. After 270 days, only 6 cases were followed for such a long period of another 270 days. The follow-up period could be ended after 250 days. As the remaining sample size for the tail period of the curve was much smaller, may not be adequate to reflect the probability distribution.
2. Overall, the sample size is not sufficient to find the survival or time to event model in the given situation. As it plays an important role in testing the hypotheses regarding the suitability of methods. In such a situation, the alternative approach is to carry out the simulation studies with varying sample sizes and test the consistency of the outcomes or results.
3. The authors did not explore time-dependent (or non-proportional hazard) scenarios in this study as the survival time distribution for non-proportional time survival will be different.
4. Authors should check the work by Gruttola and Lagakos (1989) who proposed nonparametric and weakly structured parametric methods for analysing survival data in which both the time origin and the failure event can be right- or interval-censored? Full detail can be found at [https://www.jstor.org/stable/pdf /2532030.pdf](https://www.jstor.org/stable/pdf%20/2532030.pdf)
5. Interval-censored data can be easily confused with grouped survival data. However, there is actually a fundamental difference between these two data structures although both usually appear in the form of intervals. The grouped survival data can be seen as a special case of interval-censored data and commonly mean that the intervals for any two subjects either are completely identical or have no overlapping. In contrast, the intervals for interval-censored data may overlap in any way. Because of this structural difference, statistical methods for grouped survival data are much more straightforward than those for interval-censored data. Did authors check the structure of their data and need to be clarified in the methods section?
6. The survival model to be used for such data are known and discussed below. It may help the author to prepare the data file for analysis.
7. 203 Ebola patients (who had been discharged from the hospital or received treatment and confirmed from the hospital record) were recruited in the study. It may not be correct if we take the start time as the day of recruitment in the study. The starting time or initial time of presence of EBOV as zero-day to be taken as the date of discharge from the hospital expecting on that day the EBOV virus is present in the semen. Accordingly, the day of recruitment is to be taken as the first observation for follow up of cases and the number of days to be counted from discharge day to the recruitment day as Ti (Time) for all the 203 patients. Ti should not be taken as zero, it may vary for each patient/recruited case depending on the date of discharge and day of recruitment ( in days and condition i.e. date of discharge ≤ day of recruitment) or to be taken as a result of the first day of follow up.
8. Censored (left/right)data ( δ): In this scenario, we have both left and right-censored data at a different level
i) From the day of discharge to the day of recruitments for each patient, if the EBOV positive ( δ = 0) else left-censored ( δ = -1).
ii) During the follow up of all the remaining positive cases, for each case as when tested negative ( δ = 1) i.e. right-censored and no follow up. But if positive, but lost to follow up or withdrawn at that scheduled date of lost to follow up test to be taken with time of even(T) censored value(( δ = 0), and so on.
iii) After preparing the data file, the survival model can be used to calculate the survival probability of EBOV. There are several models, but the choice model will be parametric or non-parametric. But the sample size may not be adequate to use the parametric model. However, based on the characteristics of survival data one can use and find a suitable model. The detailed procedure is available in the book noted below for reference.
9. There are three or four methods/approaches suggested in this study for fitting the survival model which may be misleading or confusing the reader as the model should be applicable to any such data or possible generalization.

Book Name: Statistics for Biology and Health: Survival Analysis

Techniques for Censored and Truncated data

By John P. Klein & Melvin L. Moeschberger

Kindly read Chapter 3 also to understand the Censoring and Truncation especially page 56 to 66.
