# Supplementary material for: Statistical methodologies for evaluation of the rate of persistence of Ebola virus in semen of male survivors in Sierra Leone
Source: PLoS One. 2022 Oct 5;17(10):e0274755. doi: 10.1371/journal.pone.0274755 (PMC9534448; doi:10.1371/journal.pone.0274755)
Supplement: Attachment — Submitted filename: Response to Reviewers.docx [file pone.0274755.s006.docx]

**PONE-D-21-05351R2**

**Title: Statistical methodologies for evaluation of the rate of**  **persistence of Ebola virus in semen of male survivors in**  **Sierra Leone**

**Comments:**

As I understand the authors want to study the survival period of EBOV (Ebola virus) in the semen of patients discharged from the hospital. In other words, it can be stated that the probability of occurrence of event X (X is negative EBOV) with the available censored data.

1. The total sample size of the study is only 203 cases and out of 203, 88 cases experienced the event (negative test of EBOV) before the day of recruitment. Only 115 cases were included for follow up study. At the same time follow up period was comparatively long i.e. till the last case was tested negative or withdrawn from the study; about 451 - 540 days. After 270 days, only 6 cases were followed for such a long period of another 270 days. The follow-up period could be ended after 250 days. As the remaining sample size for the tail period of the curve was much smaller, may not be adequate to reflect the probability distribution.

**Authors response:** For the interval censored survival estimation of EBOV persistence in semen, all the 203 male participants were followed up to the time they tested consecutively twice negative for EBOV in semen. This implies that even the 88 participants who had already experienced the event had contributed persistence data at enrolment and also at one additional post-enrolment consecutive visit; with the right-limit of the interval censoring interval set as the first of the two consecutively negative visits i.e the enrolment visit. SLEVPS team was interested also in the estimation of EBOV persistence in other body fluids, therefore all the 203 participants continued to contribute persistence data for other body fluids, even after being EBOV-free in semen. Understanding how long the persistence of the virus in semen lasts is of public health importance and this is behind using all available information on persistence even for those whose virus in the semen persisted the longest.(WHO/VHF/2018.1). We have furthermore estimated the percentiles of persistence whose figures are unaffected by the extreme observations.

2. Overall, the sample size is not sufficient to find the survival or time to event model in the given situation. As it plays an important role in testing the hypotheses regarding the suitability of methods. In such a situation, the alternative approach is to carry out the simulation studies with varying sample sizes and test the consistency of the outcomes or results.

**Authors response: Thanks for your suggestion.** The aim of the SLEVPS was to estimate the duration and rate at which the EBOV persists in semen. All recruited subjects were followed up to the time of confirmed EBOV-negative in semen); or until the censoring time (premature discontinuation or loss to follow up), counted from time of discharge from Ebola Treatment Unit (ETU). Given the nature of this outcome, we believe strongly that the survival (time-to-event) methods would be the most appropriate for this evaluation. This study was designed and implemented during an emergency setting of the EVD outbreak with the sample size selected based on convenience.The SLEVPS researchers had no control over the number of participants to recruit nor how soon to recruit following recovery (as described in the manuscript). For this paper we describe how the traditional right-censored methods and the interval-censored methods could be used and how they perform in the evaluation of EBOV persistence when massive left-censoring is present, due to the limitations in study design for Ebola persistence. We agree with the reviewer on the importance of conducting simulations, however for the current manuscript we believe that the data and results we presented in our manuscript still provide interesting insights to dealing with data of this nature.

3. The authors did not explore time-dependent (or non-proportional hazard) scenarios in this study as the survival time distribution for non-proportional time survival will be different.

**Authors response: In the current paper** we did not explore non-proportional hazards assumptions since it was not aimed at comparing survival (or hazard rates) between exposure groups. The aim of this manuscript was solely to estimate persistence (survival) among male Ebola disease survivors, using the right- and the interval-censored methods and describe their distributions.

4. Authors should check the work by Gruttola and Lagakos (1989) who proposed nonparametric and weakly structured parametric methods for analysing survival data in which both the time origin and the failure event can be right- or interval-censored? Full detail can be found at [https://www.jstor.org/stable/pdf /2532030.pdf](https://www.jstor.org/stable/pdf%20/2532030.pdf)

**Authors response: Thanks for your suggestion and** for the reference. We have looked at it. In our paper we have illustrated hthe use of both nonparametric and parametric (Weibull) interval censored models could be applied to interval- and right.-censored Ebola persistence data.

5. Interval-censored data can be easily confused with grouped survival data. However, there is actually a fundamental difference between these two data structures although both usually appear in the form of intervals. The grouped survival data can be seen as a special case of interval-censored data and commonly mean that the intervals for any two subjects either are completely identical or have no overlapping. In contrast, the intervals for interval-censored data may overlap in any way. Because of this structural difference, statistical methods for grouped survival data are much more straightforward than those for interval-censored data. Did authors check the structure of their data and need to be clarified in the methods section?

**Authors response:** Our data is not grouped sutvival data, rather it was data that was collected on individual males survivors, with a known time of origin (date of ETU discharge) and known time interval in which the event of primary interest (confirmed EBOV clearance from semen) or censoring time, for each participant. This is already clarified in the manuscript (lines 211-235).

6. The survival model to be used for such data are known and discussed below. It may help the author to prepare the data file for analysis.

**Authors response:** Thanks for this comment. The data file was already prepared and was used to run the proposed models and estimates.

7. 203 Ebola patients (who had been discharged from the hospital or received treatment and confirmed from the hospital record) were recruited in the study. It may not be correct if we take the start time as the day of recruitment in the study. The starting time or initial time of presence of EBOV as zero-day to be taken as the date of discharge from the hospital expecting on that day the EBOV virus is present in the semen. Accordingly, the day of recruitment is to be taken as the first observation for follow up of cases and the number of days to be counted from discharge day to the recruitment day as Ti (Time) for all the 203 patients. Ti should not be taken as zero, it may vary for each patient/recruited case depending on the date of discharge and day of recruitment ( in days and condition i.e. date of discharge ≤ day of recruitment) or to be taken as a result of the first day of follow up.

**Authors response:** Indeed we have taken Time 0 as the date of ETU discharge, and not the date of recruitment. Recruitment visit is the first day of post-ETU discharge follow-up.

8. Censored (left/right)data ( δ): In this scenario, we have both left and right-censored data at a different level
i) From the day of discharge to the day of recruitments for each patient, if the EBOV positive ( δ = 0) else left-censored ( δ = -1).
ii) During the follow up of all the remaining positive cases, for each case as when tested negative ( δ = 1) i.e. right-censored and no follow up. But if positive, but lost to follow up or withdrawn at that scheduled date of lost to follow up test to be taken with time of even(T) censored value(( δ = 0), and so on.
iii) After preparing the data file, the survival model can be used to calculate the survival probability of EBOV. There are several models, but the choice model will be parametric or non-parametric. But the sample size may not be adequate to use the parametric model. However, based on the characteristics of survival data one can use and find a suitable model. The detailed procedure is available in the book noted below for reference.

**Authors response:** This is noted, and t is along the lines that we have described the models proposed.

9. There are three or four methods/approaches suggested in this study for fitting the survival model which may be misleading or confusing the reader as the model should be applicable to any such data or possible generalization.

Book Name: Statistics for Biology and Health: Survival Analysis

Techniques for Censored and Truncated data

By John P. Klein & Melvin L. Moeschberger

Kindly read Chapter 3 also to understand the Censoring and Truncation especially page 56 to 66.

**Authors response:** This is noted. Thanks for your comments. We have read and refered to this book in our paper already (line 704, reference no. 13)
